# Consonant aspiration in Mandarin-speaking children: a developmental perspective from perception and production

**DOI:** 10.3389/fped.2024.1465454

**Published:** 2025-01-07

**Authors:** Yani Li, Qun Li, Yihang Du, Lili Wang, Lin Li, Jian Wen, Yun Zheng

**Affiliations:** ^1^ Department of Otolaryngology-Head and Neck Surgery, West China Hospital of Sichuan University, Chengdu, China; ^2^ Department of Audiology and Speech Language Pathology, West China Hospital, Sichuan University, Chengdu, China

**Keywords:** child development, Mandarin, consonant aspiration, perception, production

## Abstract

**Introduction:**

This study investigates Mandarin-speaking children's acquisition of aspirated/unaspirated voiceless consonants in terms of perception and production, to track children's developmental profile and explore the factors that may affect their acquisition, as well as the possible association between perception and production.

**Methods:**

Mandarin-speaking children (*N* = 95) aged 3–5 and adults (*N* = 20) participated in (1) a perception test designed based on the minimal pairs of unaspirated/aspirated consonants in the quiet and noisy conditions respectively; (2) a production test where participants produced the target words, with syllable-initial consonants focusing on aspiration and non-aspiration. Six pairs of unaspirated/aspirated consonants in Mandarin were included.

**Results:**

(1) Children's perception and production accuracy of aspirated and unaspirated consonants increased with age. Five-year-olds achieved high accuracy in the perception under the quiet condition and in the production (over 90%), though not yet adult-like. (2) Noise adversely affected children's perception, with all child groups showing poor performance in the noisy condition. In terms of perception, stops were more challenging to children than affricates, but in terms of production, children performed better on stops. Furthermore, the presence of noise had a greater detrimental effect on the perception of aspirated consonants compared to unaspirated ones. (3) A weak positive correlation was found between children's perception of consonant aspiration in the quiet condition and their production.

**Discussion:**

The findings indicate that age, aspiration state, and manner of articulation (MOA) would affect children's acquisition of consonant aspiration. Although 5-year-olds have almost acquired aspirated/unaspirated consonants, compared to adults, the perception of consonant aspiration in noise remains a challenge for children.

## 1 Introduction

Consonants, constituting approximately 58.5% of speech sounds, are pivotal to speech intelligibility and clarity (1, 2). Aspiration is an important distinctive feature of consonants that divides stops and affricates into aspirated and unaspirated sounds (3). This feature, defined as the turbulent airflow generated near the vocal folds following the burst release of these sounds, is found in many languages such as English, Mandarin, and the Indic languages (3, 4). In English, aspiration is considered a phonetic feature of voiceless stops. For example, the phoneme /k/ appears as aspirated /k^h^/ in “kit” (/k^h^ɪt/) and unaspirated /k/ in “skit” (/skɪt/). Although /k^h^/ and /k/ differ phonetically, they do not entail phonemic contrasts, as the presence or absence of aspiration will not change the meaning of words. Thus, these different sounds are nonetheless considered to belong to the same phoneme in English. In contrast, Mandarin features six pairs of aspirated/unaspirated consonants, including three pairs of stops (/p/-/p^h^/,/t/-/t^h^/,/k/-/k^h^/) and three pairs of affricates (/tɕ/-/tɕ^h^/,/ʦ/-/ʦ^h^/,/ʈʂ/-/ʈʂ^h^/). These pairs are phonemically distinct and function as separate phonemes in Mandarin, carrying semantic weight. For instance, /tu/ (tone 4, unaspirated) means “belly”, while /t^h^u/ (tone 4, aspirated) means “rabbit”. If a speaker used /t/ instead of the /t^h^/, the meaning of the word would change from “rabbit” to “belly”. Therefore, the perception and production of these aspirated/unaspirated contrasts are crucial for children in the development of Mandarin.

Aspiration can be quantified by voice onset time (VOT), the interval between the burst's release and vocal fold vibration. VOT serves as a critical perceptual cue and an objective manifestation of aspiration production (5), with aspirated consonants exhibiting a longer VOT than their unaspirated counterparts. Young children exhibit low sensitivity to static cues like duration (6, 7), which may contribute to the delayed acquisition of aspiration. The physiological complexity involved in controlling aspiration contrasts may further contribute to this challenge. Moreover, children with hearing loss may struggle with speech intelligibility, making it difficult for them to accurately perceive and produce consonants, leading to distorted aspiration contrasts (8). To learn children's acquisition of consonant aspiration and provide developmental milestones for speech therapy, especially for children with hearing loss, this study investigates Mandarin-speaking children's perception and production of consonant aspiration from a developmental perspective, and explores the factors that may affect children's acquisition.

Previous research has shown that children's accuracy in perceiving consonant aspiration improves with age. Using syllable recognition tasks that present aspirated and unaspirated consonants as minimal pairs in natural speech, Liu et al. (9) found that most Mandarin-speaking children had acquired the perception of aspirated and unaspirated consonant pairs by age 3, with the /k/-/k^h^/ pair being the last to develop. However, the live presentation of voice stimuli by testers might have provided visual cues, potentially overestimating children's perception abilities. Wong et al. (10) employed pre-recorded adult speech and a simple discrimination task, finding that children around 5;8 years old achieved over 90% accuracy in the quiet condition, with performance close to adults by 6;5 years. Specifically, children in their study developed the ability to discriminate between contrasts such as /ʦ/-/ʦ^h^/,/t/-/t^h^/, and /k/-/k^h^/ relatively earlier, with /p/-/p^h^/ being acquired last, around ages 6;5–7;6. Despite these findings, the specific developmental trajectory of aspiration perception remains unclear due to variations in task designs and participant age ranges across studies. This calls for a systematic reevaluation of the development of perceiving consonant aspiration by Mandarin-speaking children.

Speech perception in children is a complex process, particularly in noisy environments that are common in daily life. Noise can significantly interfere with accurate speech perception, an issue that is exacerbated for children due to their developing auditory systems being more susceptible to masking effects (11). Children's developing auditory systems, coupled with limited language experience and evolving cognitive abilities, make it challenging for them to extract critical speech information from background noise, leading to delayed acquisition of perception skills in the noisy condition (12). It is not until after the age of ten that English-speaking children's ability to recognize consonants in noise approaches that of adults (13). Similarly, Wong et al. (10)'s study on Mandarin consonant aspiration discrimination in children aged 4;0–8;9 under speech-shaped noise (SSN) at various signal-to-noise ratios (SNRs) revealed that while most children aged 7;8–8;9 could achieve over 75% correct discrimination at 0 and −5 dB SNRs, they had not yet demonstrated adult-like performance. Based on these findings, it can be suggested that the perception of aspirated and unaspirated consonants in noise may develop significantly later than in the quiet condition, potentially not maturing until after the age of ten. However, the majority of research has concentrated on the challenges faced by children with hearing impairments in noisy environments (14, 15). Furthermore, previous studies have often included various consonant contrasts without specifically addressing the perception of aspiration in noisy environments. Consequently, the perception of aspirated and unaspirated consonants in noise by Mandarin-speaking children with normal hearing, particularly among early language learners, remains unclear. To address this gap, the present study employs a comprehensive set of aspirated/unaspirated minimal pairs to explore the effects of noise on Mandarin-speaking children's speech perception. Given the widespread application of SSN due to its spectral coverage of most speech frequencies (10, 16, 17), this study also utilizes SSN as the noise environment. Additionally, to provide a sensitive measure of speech perception abilities in noise and to avoid floor effects (18–20), the study selects an SSN masker at 0 dB SNR. This choice is also intended to simulate the auditory conditions of a typical real-world or classroom environment, creating a moderately challenging listening scenario for children (21).

In addition, it is well known that children's perception of consonants is influenced by the acoustic characteristics of consonants. For instance, aspirated consonants have longer VOT values and stronger airflow noise than their unaspirated counterparts (22). Research indicates that children typically acquire shorter VOT patterns earlier, suggesting that unaspirated consonants may be perceived more accurately and early than aspirated ones (23). As such, the aspiration state of consonants may be a contributing factor to children's perceptual performance. Furthermore, the manner of articulation (MOA) also affects the perception of consonant aspiration, such as stops and affricates. Stops consist of a closure phase and a release phase, with the distinction of aspiration features relying primarily on VOT. In contrast, affricates, which involve a closure phase, a release phase, and an additional frication phase, require both VOT and fricative noise to distinguish aspirated and unaspirated affricates, making them perceptually more complex than stops (24, 25). While the additional frication cues in affricates increase perceptual complexity for children (26), they also provide greater resistance to masking in noisy environments compared to VOT (27, 28). This distinction underscores the necessity of considering MOA when examining children's perception of aspirated and unaspirated consonants. By examining the perception of aspirated and unaspirated stops and affricates in the quiet and noisy conditions, this study aims to clarify how different acoustic and articulatory factors influence children's perceptual accuracy.

Similar to perception development, the development of children's production of aspirated and unaspirated voiceless consonants is strongly influenced by age and involves intricate articulatory and motor skills. In early motor speech development, children require more time to master aspirated consonants due to the need for precise control over vocal timing, particularly in delaying laryngeal vibration during the release of oral closure (29). While unaspirated consonants involve almost immediate vocal fold vibration after oral release, aspirated consonants require more complex coordination, including glottal timing, sustained airflow, and delayed vocal fold vibration (22, 30). Research on Mandarin-speaking children shows that unaspirated consonants, characterized by shorter VOTs, are typically acquired by age 3, whereas aspirated consonants, with longer VOTs, remain less stable until around age 6, with notable individual variation (30). These findings are similar to studies in other languages, such as English, where short-lag VOTs for unaspirated stops are generally mastered by age 2, while long-lag VOTs for aspirated stops take longer to develop (29, 31). The developmental trajectory of VOT in children, however, shows considerable variability. Some studies report shorter VOTs in children aged 4–6 compared to adults (29, 32), while others find longer VOTs within the same age group (30, 33, 34). For instance, Ma et al. (33) studied Mandarin-speaking children aged 6–15 and found that 6- to 7-year-olds had longer VOTs than adults for highly aspirated stops, with 7- to 8-year-olds showing VOT patterns similar to adults. More importantly, the exact developmental profile of consonant aspiration acquisition remains unclear. While objective measures such as VOT from previous studies provide valuable insights related to physiological development, they do not offer definitive markers of when the skill is fully acquired. VOT measurements alone may not fully capture whether children's production of aspirated and unaspirated consonants results in perceptible phonemic contrasts that are recognizable to native listeners in everyday communication (35, 36). In real-world contexts, the perceptual judgments of native speakers are crucial for determining whether children have successfully acquired these sounds (35, 37). Therefore, this study aims to investigate whether the aspirated and unaspirated consonants produced by children form distinct phonemic contrasts that can be accurately identified by native listeners, thus supporting effective communication. Additionally, considering the varying levels of articulatory difficulty between aspirated and unaspirated sounds, the study also examines the impact of aspiration state on production accuracy.

The MOA also plays a critical role in children's production of aspirated and unaspirated consonant contrasts. Cross-linguistic studies on speech acquisition suggest that stops, which involve a straightforward complete closure and release, are typically acquired earlier than other consonant types, typically by age 4 (38, 39). In contrast, affricates require a more complex articulation process, involving both a complete closure and a rapid release followed by a narrow constriction to produce a slow, frictional airflow. This complexity makes affricates more challenging for young children, who frequently substitute stops for affricates, particularly among children aged 2–4 years (40). Ma et al. (41) found that Mandarin-speaking children aged 3–5 often replaced one type of affricates with another, indicating that while children may differentiate affricates from stops, they still face challenges in precisely distinguishing different affricate sounds (42). Given these complexities, it can be suggested that preschool children's ability to produce stable contrasts between aspirated and unaspirated consonants is influenced not only by aspiration state but also by MOA, with stops potentially exhibiting more stable production patterns than affricates. However, previous research on the production of aspirated and unaspirated consonants in children has largely focused on stops (43, 44), leaving a gap in understanding how children develop the ability to produce affricates with varying aspiration features. To address this, the present study includes both stops and affricates to examine whether the distinct developmental patterns hold across these two consonant types in terms of producing aspiration contrasts.

Building on the identified research gaps, this study examines the perception and production of aspirated and unaspirated consonants in preschool children. Effective language communication requires the ability to both perceive and produce the phonemic contrasts of a language, yet the interplay between perception and production remains a perennial issue in speech acquisition research (45). While first language acquisition studies have shown that infants can perceive many phonetic contrasts before they can produce them accurately (46, 47), whether this tight association holds for phonemic distinctions such as consonant aspiration in Mandarin is still underexplored.

Previous research on children's development of consonants has often focused on either the perception or production of aspirated and unaspirated contrasts, making direct comparisons challenging. As a result, the literature presents an unclear picture of the relationship between consonant aspiration perception and production in children. Some recent studies have begun to explore both abilities, but the findings are mixed. Shultz et al. (48) found a non-significant positive trend between the perception of the /b/-/p/ continuums and VOT production patterns in 32 English native speakers. Mahshie et al. (49) reported a moderate correlation (*r* = 0.57, *p* = .02) between perception and production for /d/-/t/ voicing contrasts in 15 English-speaking preschool children with cochlear implants. In addition, Idemaru et al. (50) and McAllister Byun et al. (51) also examined the English /r/-/l/ and /r/-/w/ contrasts respectively, with mixed results.

The observed instability in the correlation between speech perception and production may, in part, stem from limitations in sample size. Studies with smaller cohorts, such as Wong's research involving only 13 Mandarin-speaking (52) and 20 Cantonese-speaking children (53), all aged 3, have reported that there was no significant link between perception and production abilities. These null findings could be attributed to a lack of statistical power. When the sample size was increased to encompass a wider age range of 4–6 years old (*N* = 48), a weak correlation was observed between Cantonese tone perception and production (*R*^2^ = 0.194) (54). Similarly, Mok et al. (55) found a weak but present correlation between perception and production in a larger dataset of 111 children. These findings underscore the critical role of an adequately sized dataset in robustly examining the relationship between speech perception and production, as well as the necessity of scrutinizing a larger and more diverse group to bridge the existing research gap.

In a nutshell, we focus on a prominent phonemic feature in Mandarin, consonant aspiration, aiming to address three main questions: (a) What is the developmental trend of consonant aspiration perception and production in 3- to 5-year-old children? (b) What factors might influence the acquisition of aspirated/unaspirated contrasts? (c) How are perception and production of consonant aspiration related?

On the basis of previous research on children's acquisition of consonant aspiration reviewed above, we predict that noisy conditions will significantly hinder children's perception of consonant aspiration (12, 13). We also expect children's accuracy in perceiving and producing aspirated and unaspirated sounds will vary according to their MOA, with expected differences in children's performance on aspirated vs. unaspirated consonants (23, 26, 42). However, the specific performance of perception and production in preschool children, especially across different consonant types (e.g., stops and affricates) and environmental conditions (quiet vs. noise), remain unclear. In addition, building on existing findings regarding the relationship between speech perception and production, we hypothesize a positive correlation between children's perception and production abilities for Mandarin aspiration features, particularly with the extended sample size in this study (54, 55).

To explore the developmental trajectory of Mandarin-speaking children's perception and production of aspirated and unaspirated consonant contrasts, and to validate our hypotheses about their association and influencing factors, we conducted a study with 95 children aged 3;3–5;11. This study includes two experiments: Experiment 1 evaluates children's perception of aspirated/unaspirated contrasts under quiet and noisy conditions to evaluate the impact of noise on perception. Experiment 2 assesses their production accuracy for these consonants in the quiet condition. By analyzing the developmental patterns in children's acquisition of consonant aspiration and identifying potential facilitators and barriers in this process, we aim to provide valuable insights that could enhance clinical practices, informing the development of targeted intervention strategies for children who struggle with speech acquisition (e.g., children with hearing loss).

## 2 Study 1: consonant aspiration perception

### 2.1 Methods

This study was approved by the Research Ethics Committee at West China Hospital of Sichuan University (No. 2023–2376).

#### 2.1.1 Participants

Ninety-five Mandarin-speaking children (48 boys and 47 girls) aged 3;3–5;11 participated in this study. They were Mandarin native speakers without any developmental disorders reported. The child subjects were divided into 3 age groups, i.e., 3-year-old group (3y), 4-year-old group (4y), and 5-year-old group (5y), as detailed in Table 1. All children passed hearing screening using otoacoustic emissions (OAE) technology (56). Furthermore, they were administered age-appropriate language test (Muyan Speech Wisdom) (57) and IQ test (Primary Test of Nonverbal Intelligence) (58), and all scored within the normal range in the language and IQ tests. In addition, 20 adult native speakers (10 males and 10 females) with unremarkable developmental history were recruited.

**Table 1:** Demographic information of the participants.

		Age (year)			
Group	Number	Mean (SD)	Range	Male	Female
Children	95	4;8 (0;11)	3;3–5;11	48	47
3y	32	3;8 (0;3)	3;3–3;11	12	20
4y	33	4;6 (0;4)	4;0–4;11	20	13
5y	30	5;4 (0;4)	5;0–5;11	16	14
Adults	20	23;4 (1;2)	19;10–24;5	10	10

#### 2.1.2 Materials

In this study, target words are monosyllabic or disyllabic words whose aspirated/unaspirated consonants differ in syllable-initial position, while the vowels and tones are the same.

The perception test is a picture selection task that consists of 48 monosyllabic words in total (6 consonant pairs × 4 vowels). The target words were selected from the “Vocabulary Frequency Reduction List for 3- to 6-year-old Mandarin-speaking Children”, “Basic Vocabulary List for 4-year-old children” and “Common Vocabulary List for 5-year-old Children in Chinese” (59), and the vocabulary list from the book “A Study on the Acquisition of Content Words in Han Children” (60). These words were further validated through pilot tests on 12 Mandarin-speaking pre-school children to ensure familiarity and appropriateness for the target age group.

**Figure 1:**
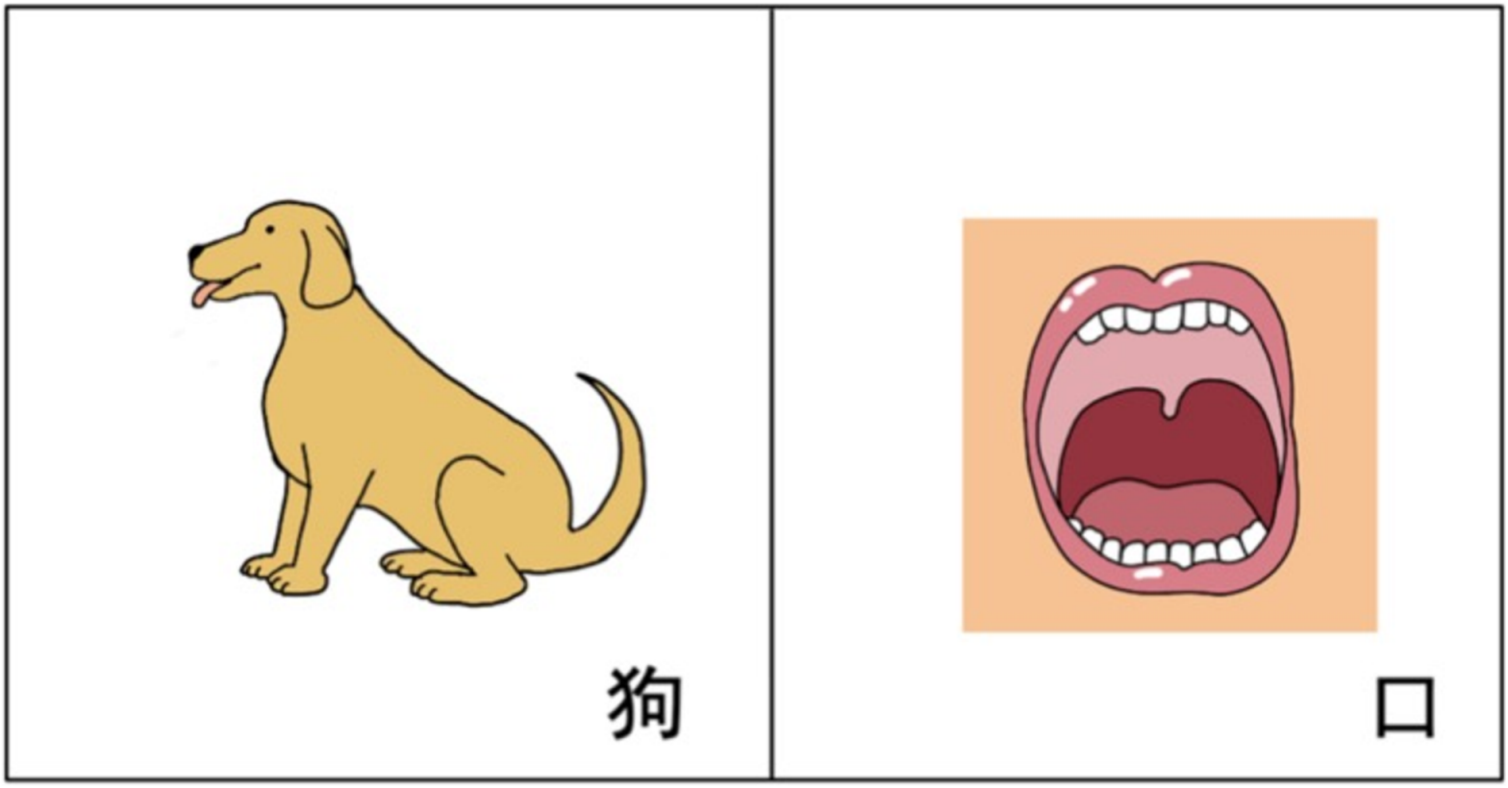
Example of the perception test based on mandarin aspirated/unaspirated consonants. The pictures represent the contrast between /k/ (left, *gou3*/koʊ/, ‘狗dog’) and /k^h^/ (right, *kou3*/k^h^oʊ/, ‘口mouth’).

Each test item in the perception task included two pictures, with one presenting the target word and the other one representing a word that formed a minimal pair (aspirated/unaspirated) with the target word (please refer to Supplementary Material A for all the stimuli). As shown in Figure 1, the target word is *gou3*/koʊ/ (狗‘dog’) and the distractor is *kou3*/k^h^oʊ/ (口‘mouth’), which shares the same vowel (/oʊ/) and tone (Tone 3) with the target word, but differs in syllable-initial consonant in terms of aspiration. The target words were presented with the same question *na3 yi1 ge4 shi4______?* (‘哪一个是____? Which one is ______?’) to keep the preceding context the same. The corpus consists of separate recordings by male and female announcers, with their voices evenly distributed across the test items. The recording took place in a double-layer soundproof room with ambient noise levels below 20 dB(A). The sound samples were recorded and edited using Adobe Audition 2022. The recording parameters were set to a single channel, a sampling rate of 44.1 kHz, sampling precision of 32-bit.

To conduct the perception test under a noisy condition, MATLAB software was used to filter white noise into SSN matching the average speech spectrum of the recordings. Then, the root mean square (RMS) amplitude of the SSN was adjusted to match the RMS amplitude of the syllable, achieving the signal-to-noise ratio (SNR) of 0 dB. The SSN was pre-mixed with the stimulus before auditory presentation, and the combined stimuli were presented through a single audio channel.

#### 2.1.3 Procedures

Before conducting the tests, written informed consent was obtained and the parents filled out a questionnaire about the demographic information and language background of the children. The conditions required for a consonant aspiration perception test included a quiet room (background noise <35 dB), a computer, and a trained researcher. Audio stimuli were presented binaurally via a laptop computer in mono at an average of 65 dB SPL.

Before the perception tests, two practice trials unrelated to the test content were used to familiarize the participants with the two-alternative forced-choice (2-AFC) paradigm. The participants faced a computer monitor and indicated the picture that corresponded to the target word they heard. In the perception task, the stimuli were first played under the noisy condition by researchers, followed by a rest period, and then under the quiet condition. The target stimuli were randomized in each listening condition to mitigate potential order effects.

Data collection in this experiment involved recording each response as a binary outcome (correct or incorrect) for each trial. From these raw data, we derived perception accuracy for each participant under each experimental condition. Perception accuracy was calculated as the proportion of correct responses and served as the dependent variable for our subsequent statistical analyses.

### 2.2 Results

To evaluate the developmental trajectory of consonant aspiration perception in children and the influence of listening conditions, we analyzed perception accuracy in children aged 3–5 under quiet and noisy conditions and compared these findings with adult performance.

**Figure 2:**
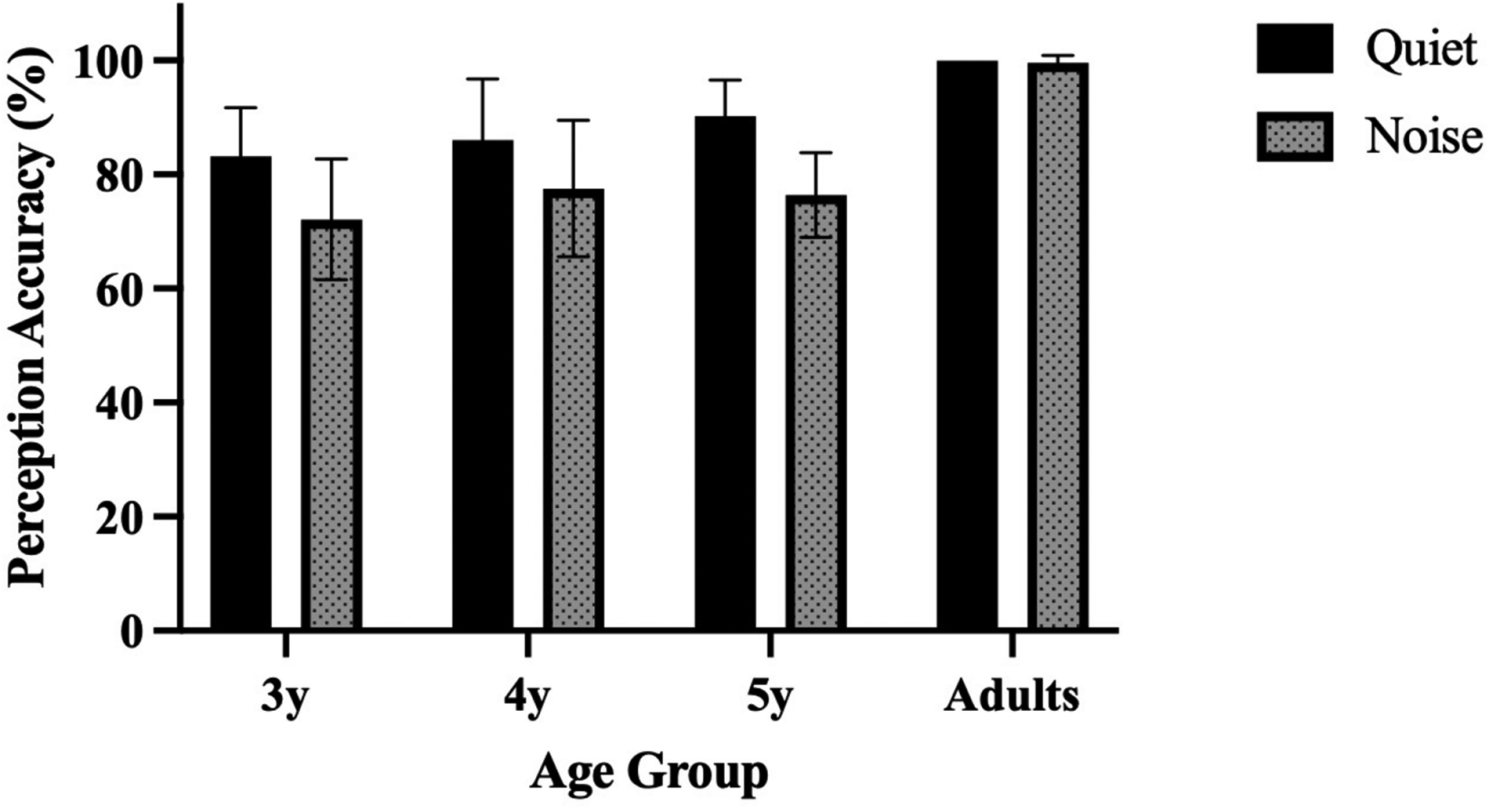
Perception of aspirated/unaspirated consonants across age groups under the quiet and noisy conditions. Error bars represent ± 1 standard deviation. 3y = 3-year-old group, 4y = 4-year-old group, 5y = 5-year-old group.

Adult participants demonstrated ceiling-level perception accuracy for consonant aspiration in both quiet (average accuracy: 100.0%, SD = 0.0%) and noisy conditions (average accuracy: 99.58%, SD = 1.3%), indicating that the test materials, such as the recordings and pictures, were clear enough for accurate judgment. The overall mean perception accuracy by age can be found in Figure 2. In the quiet condition, the average perception accuracy for 3-year-olds, 4-year-olds, and 5-year-olds was 83.2% (SD = 8.5%), 86.0% (SD = 10.8%), 90.3% (SD = 6.3%), respectively, showing an age-related improvement. In the noisy condition, the average accuracy was lower: 72.1% (SD = 10.6%), 77.5% (SD = 12.0%), and 76.4% (SD = 7.4%) for 3-, 4-, and 5-year-olds, respectively. One-sample *t*-tests indicated that all age groups significantly exceeded chance level (1/2 = 50%), *p* < .001.

To substantiate the effects of age and listening conditions on consonant aspiration perception, a two-way mixed ANOVA was conducted using R statistical software (61). The analysis included *Age Group* (3-year-old, 4-year-old, and 5-year-old children and adults) and *Condition* (quiet, noise) as fixed factors, revealing significant main effects for *Age Group*, *F*(3, 111) *=* 40.100, *p* < .001, *η*^2^ = 0.519, and *Condition*, *F*(1, 111) *=* 110.675, *p* < .001, *η*^2^ = 0.699, and a significant interaction between them, *F*(3, 111) *=* 8.834, *p* < .001, *η*^2^ = 0.193. To further explore this interaction, post-hoc analyses were conducted using the emmeans package in R with Tukey adjustments for multiple comparisons. These comparisons examined differences within age groups across conditions (e.g., “Quiet 3y vs. Noise 3y”) and across age groups within each condition (e.g., “Quiet Adults vs. Quiet 3y”), allowing us to access developmental changes in perception accuracy under varying listening conditions (see Supplementary Material C1 for full statistical details).

For child participants, there was a significant improvement in the quiet condition between ages 3 and 5 (*p* = .034). However, in the noisy condition, performance across age groups showed little difference (*p* > .05). In both conditions, children's perception accuracy was significantly lower than that of adults (*p* < .001). These results indicate that although 5-year-olds show high accuracy in the quiet condition (above 90%), they have not yet reached adult-like performance. In the noisy condition, the minimal improvement from ages 3–5 indicates that children have not fully developed the perceptual skills necessary for reliable aspiration perception in adverse listening conditions.

**Figure 3:**
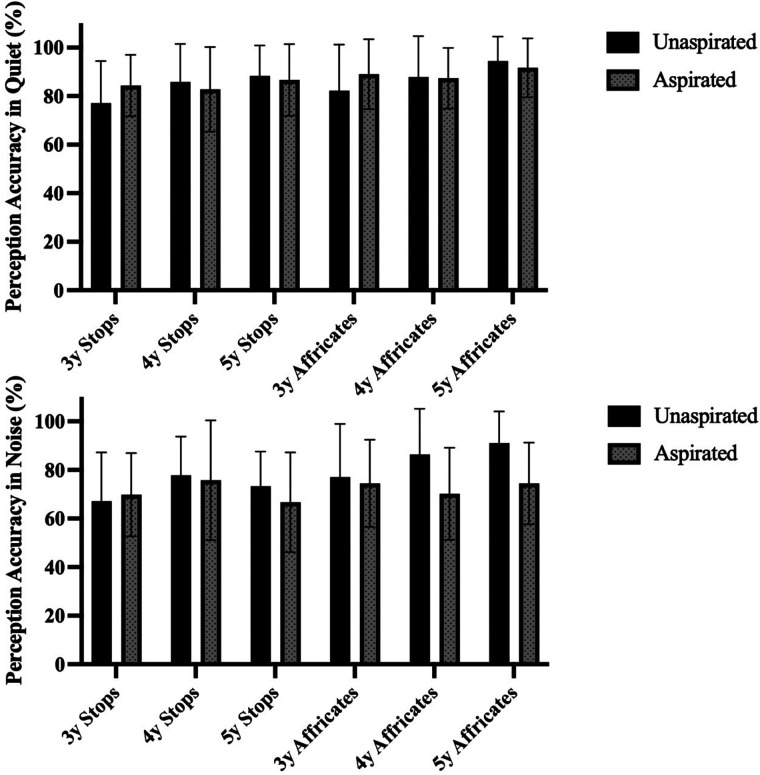
Children's perception of aspirated/unaspirated consonants (stops and affricates) in the quiet and noisy conditions. Error bars represent ± 1 standard deviation. 3y = 3-year-old group, 4y = 4-year-old group, 5y = 5-year-old group.

To explore factors that may affect children's perceptual performance on consonant aspiration, this study analyzed their ability to perceive aspirated and unaspirated stops and affricates under various conditions. Figure 3 summarizes children's perception accuracy across these conditions. Overall, age influenced perception, except for the perception of aspirated stops and affricates in noise, which aligns with the trends shown in Figure 2. The clear advantage in the quiet condition performance was present in all children, but notably, unaspirated consonants were less affected by noise than aspirated consonants, particularly unaspirated affricates. While the differences in perception accuracy between aspirated and unaspirated consonants were generally small across age groups, 4- and 5-year-olds showed over 10% higher accuracy for unaspirated affricates compared to aspirated ones in the noisy condition. Additionally, affricates were generally perceived more accurately than stops in noise, especially unaspirated affricates.

To further substantiate the influence of these factors on children's perception, two generalized linear mixed-effects models (GLMMs) were constructed respectively. The first model (hereafter referred to as *GLMM1*) included *Condition*, *Aspiration State* (aspirated, unaspirated), *MOA* (stops, affricates), and their interactions as fixed effects, while the second model (*GLMM2*) incorporated *Child Age* (3-year-old, 4-year-old and 5-year-old children), *Aspiration State*, *MOA*, and their interactions as fixed effects. Both models included random intercepts by participant. Initially, the models included random intercepts and slopes (62), but convergence issues indicated that the model was overly complex. As a result, only random intercepts were retained to ensure model stability. Post-hoc pairwise comparisons were performed using the emmeans package with Tukey adjustment.

The results from *GLMM1*, as detailed in Table 2, demonstrated significant effects of the *Aspiration State* and *MOA* of consonants and *Condition* on children's perception, with significant interaction effects among these factors, except for the *Condition* × *MOA* interaction. Post-hoc comparisons (detailed in Supplementary Material C1) further clarified these interactions, revealing significant effects for *Condition* × *Aspiration State*, *MOA* × *Aspiration State*, and *Condition* × *Aspiration State* × *MOA*.

**Table 2:** The results of GLMM1 for children's perception accuracy (*p < .05, **p < .01, ***p < .001).

Fixed Effects	*χ* ^2^	Df	*p*	
(Intercept)	278.778	1	<.001	***
Condition	18.328	1	<.001	***
Aspiration state	6.141	1	.013	*
MOA	24.226	1	<.001	***
Condition: Aspiration state	11.373	1	<.001	***
Condition: MOA	1.109	1	.292	
Aspiration state: MOA	4.117	1	.042	*
Condition: Aspiration state: MOA	4.747	1	.029	*

The two-way interaction analysis of *Condition* × *Aspiration State* showed that the effect of the *Aspiration State* (presence vs. absence of aspiration) depended on the *Condition* (presence vs. absence of noise). In the quiet condition, children performed similarly in perceiving aspirated and unaspirated consonants. However, in the noisy condition, their perception accuracy of aspirated consonants was significantly lower than that of unaspirated ones (*p* < .001). The analysis of the *MOA* × *Aspiration State* interaction revealed that the effect of the *Aspiration State* also varied with *MOA* (stops vs. affricates). For affricates, unaspirated tokens were perceived more accurately than aspirated ones (*p* = .008), while no significant difference was observed between aspirated and unaspirated stops (*p* = .989). Additionally, for unaspirated consonants, affricates were perceived more accurately than stops (*p* < .001). Further analysis of the three-way interaction revealed that the effect of the *Condition* was influenced by both *Aspiration State* and *MOA*. Noise significantly impaired the perception of aspirated stops, unaspirated stops, and aspirated affricates (*p* < .001), while the perception of unaspirated affricates remained relatively stable across the quiet and noisy conditions (*p* = .843). In the noisy condition, unaspirated affricates were perceived significantly more accurately than both unaspirated stops and aspirated affricates (*p* < .001). These findings collectively highlight the perceptual advantage of unaspirated cues and affricates, particularly under the noisy condition. The results underscore the importance of considering the interplay among listening conditions, aspiration state, and MOA to better understand children's consonant perception patterns.

*GLMM2*, as shown in Table 3, indicated significant effects of the *Aspiration State* and *MOA* of consonants and *Child Age* on children's perception, with a significant interaction between *Aspiration State* and *Child Age*. Post-hoc comparisons (detailed in Supplementary Material C1) showed that 4-year-old (*p* < .05) and 5-year-old (*p* < .05) children demonstrated superior perception of unaspirated consonants compared to aspirated ones, while 3-year-olds showed no significant difference (*p* > .05). Furthermore, 3-year-old children's perception of unaspirated consonants was significantly lower than that of 4-year-olds (*p* < .05) and 5-year-olds (*p* = .05). However, perception accuracy for aspirated consonants remained similar across all age groups (*p* > .05). These findings underscore the earlier developmental progress in children's perception of unaspirated consonants relative to aspirated consonants.

**Table 3:** The results of GLMM2 for children's perception accuracy (*p < .05, **p < .01, ***p < .001).

Fixed Effects	*χ* ^2^	Df	*p*	
(Intercept)	82.068	1	<.001	***
Child age	18.064	2	<.001	***
Aspiration state	4.489	1	.034	*
MOA	17.189	1	<.001	***
Child age: Aspiration state	10.853	2	.004	**
Child age: MOA	1.942	2	.379	
Aspiration state: MOA	2.904	1	.088	
Child age: Aspiration state: MOA	0.233	2	.890	

## 3 Study 2: consonant aspiration production

The 95 children and 20 adults who took part in the perception task also participated in the consonant production task.

### 3.1 Methods

#### 3.1.1 Materials

**Figure 4:**
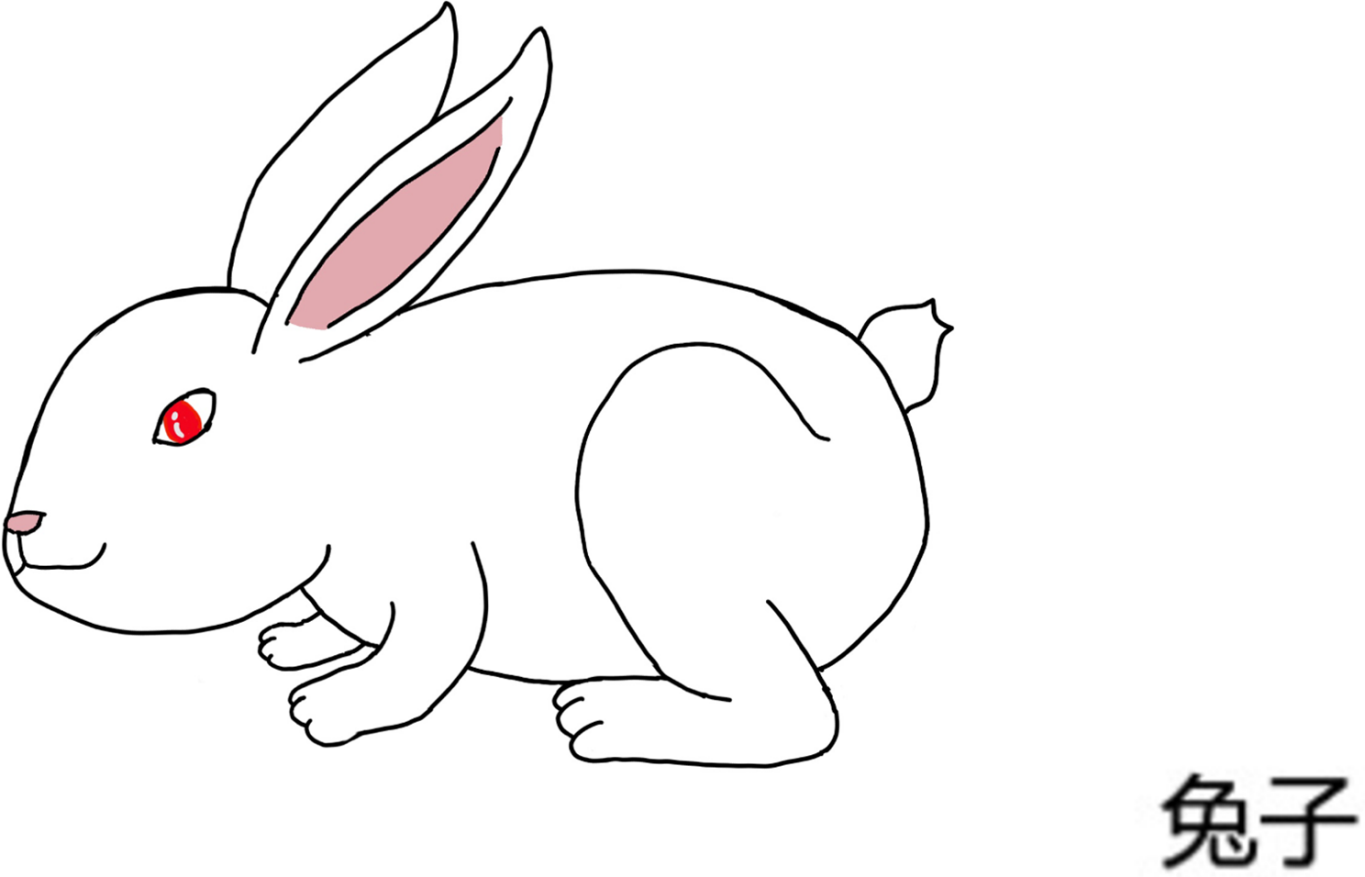
Example of the production test based on Mandarin aspirated/unaspirated consonants. Picture used to elicit /t^h^/ in *tu4 zi*/t^h^u ʦz̩/ (兔子‘rabbit’).

The production test consists of 48 disyllabic words in total (6 consonant pairs × 4 vowels). Participants have to name the pictures that represent the 48 target disyllabic words, with the syllable-initial aspirated or unaspirated consonants (please refer to Supplementary Material B for all the target words). As shown in Figure 4, the target word of this picture is *tu4 zi*/tʰu ʦz̩/ (兔子‘rabbit’), where the target consonant is the syllable-initial consonant /t^h^/ of the first syllable. Instruction for the production task was also pre-recorded by announcers, the same as the perception test, in order to ensure that all subjects followed the same instructions.

#### 3.1.2 Procedures

The conditions required for the aspirated/unaspirated consonants production test are the same as those in the perception test, with the addition of a voice recorder (iFLYTEK H1 Pro). Again, two practice trials were used to familiarize the participants with the picture naming task. Pictures were displayed on a computer, and participants were encouraged to spontaneously produce the words via a prompt question from the researcher, such as *zhe4 shi4 shen2 me?* (‘这是什么? What is this?’). If participants failed to produce the target word, they were asked to repeat the pre-recorded adult speech. The entire process was recorded, and the digital voice recorder (mono, 16-bit precision, 44.1 kHz sampling rate) was placed approximately 10–15 cm from the subject's mouth.

#### 3.1.3 Data analysis

In the production task, each subject produced 48 disyllabic words that contained the target aspirated/unaspirated consonants. A total of 4,560 (48 × 95) speech samples from the 95 children and 960 (48 × 20) speech samples from the 20 adults were recorded. However, two children's speech samples were excluded from the analysis due to poor recording quality. Therefore, the final dataset included 4,464 (48 × 93) speech samples from 93 children and 960 (48 × 20) speech samples from 20 adults.

Two trained research assistants, both native speakers of Standard Mandarin with expertise in audiology and speech-language pathology, participated in the analysis of participants’ recordings of aspirated/unaspirated consonants. They independently judged the production of target consonants as either correct or incorrect. Inter-rater reliability was high (ICC = 0.908, *p* < .001), indicating excellent consistency between the two research assistants. Production accuracy was calculated as the proportion of correct responses across trials for each participant. The final production accuracy scores were calculated as the average of the two assistants’ assessments and served as the dependent variable in the subsequent statistical analyses. In addition to analyzing production accuracy, an error analysis was conducted to explore specific patterns of production errors. Errors were further classified following To et al. (63) into categories such as POA confusion (e.g., fronting, backing), aspiration confusion (e.i., deaspiration, aspiration), MOA confusion (e.g., stopping, affrication), and consonant deletion (e.g., replacing /pa/ with /a/). The percentage of each error type relative to the total number of incorrect productions was also calculated.

### 3.2 Results

**Figure 5:**
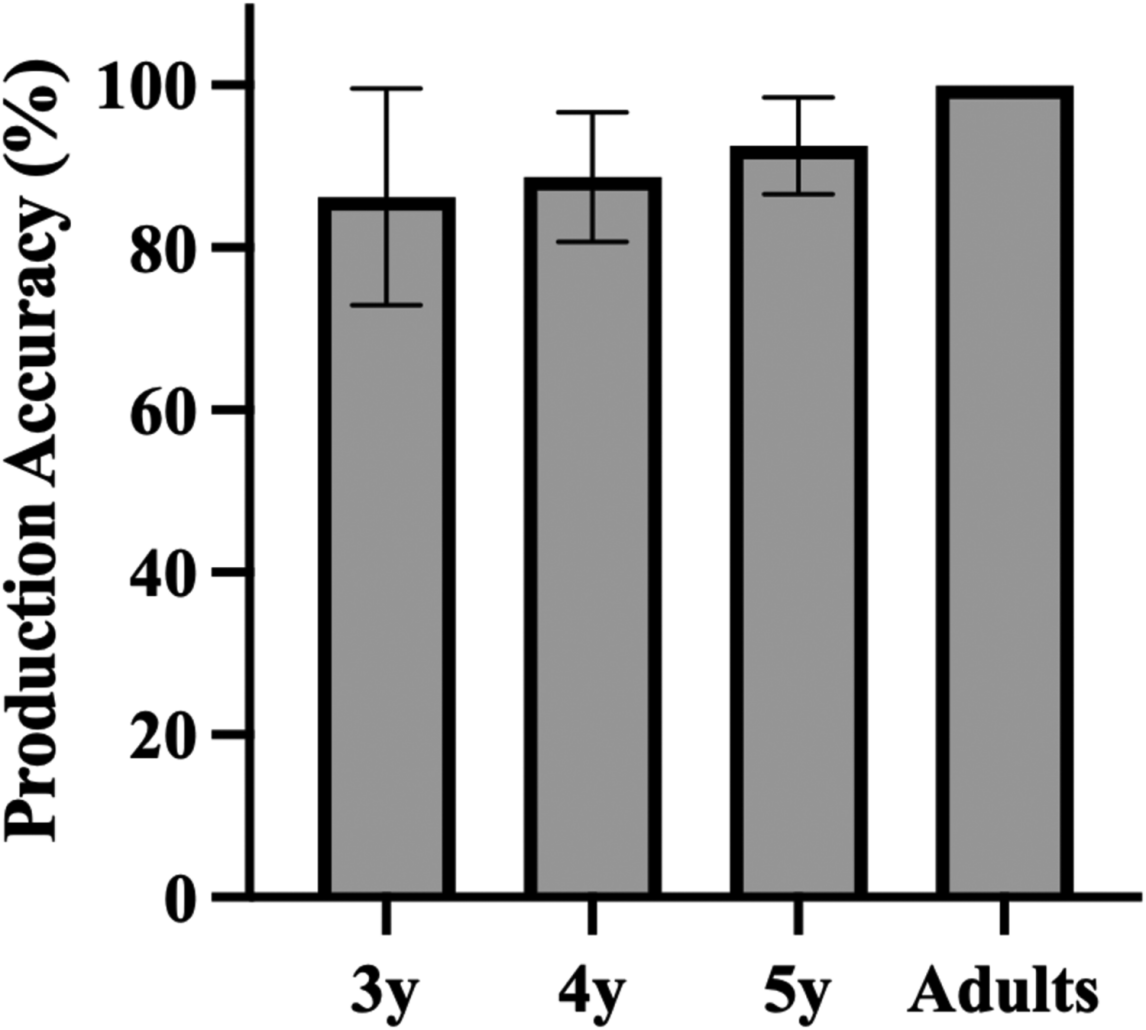
Accuracy of producing aspirated/unaspirated consonants in children across age groups and adults. Error bars represent ± 1 standard deviation. 3y = 3-year-old group, 4y = 4-year-old group, 5y = 5-year-old group.

To assess the developmental progress in the production accuracy of aspirated and unaspirated consonants, this study also analyzed the production accuracy of 3- to 5-year-old children and compared it with that of adults. Adult participants performed perfectly in producing both aspirated and unaspirated consonants. Figure 5 illustrates the average production accuracy for 3-year-olds, 4-year-olds, and 5-year-olds, which was 86.3% (SD = 13.3%), 88.6% (SD = 7.9%), and 92.6% (SD = 6.0%), respectively. A one-way ANOVA with *Age Group* as the factor revealed a significant main effect, *F*(3, 109) *=* 13.630, *p* < .001, *η*^2^ = 0.273. Comparisons showed significant differences in production accuracy between adults and each age group of children (*p* < .001). However, no significant differences were found among the children's age groups (*p* > .05; further details in Supplementary Material C2). These findings suggest that while 5-year-olds exhibited good ability to produce the aspirated/unaspirated consonants (over 90%), their performance still fell short of adult levels.

**Figure 6:**
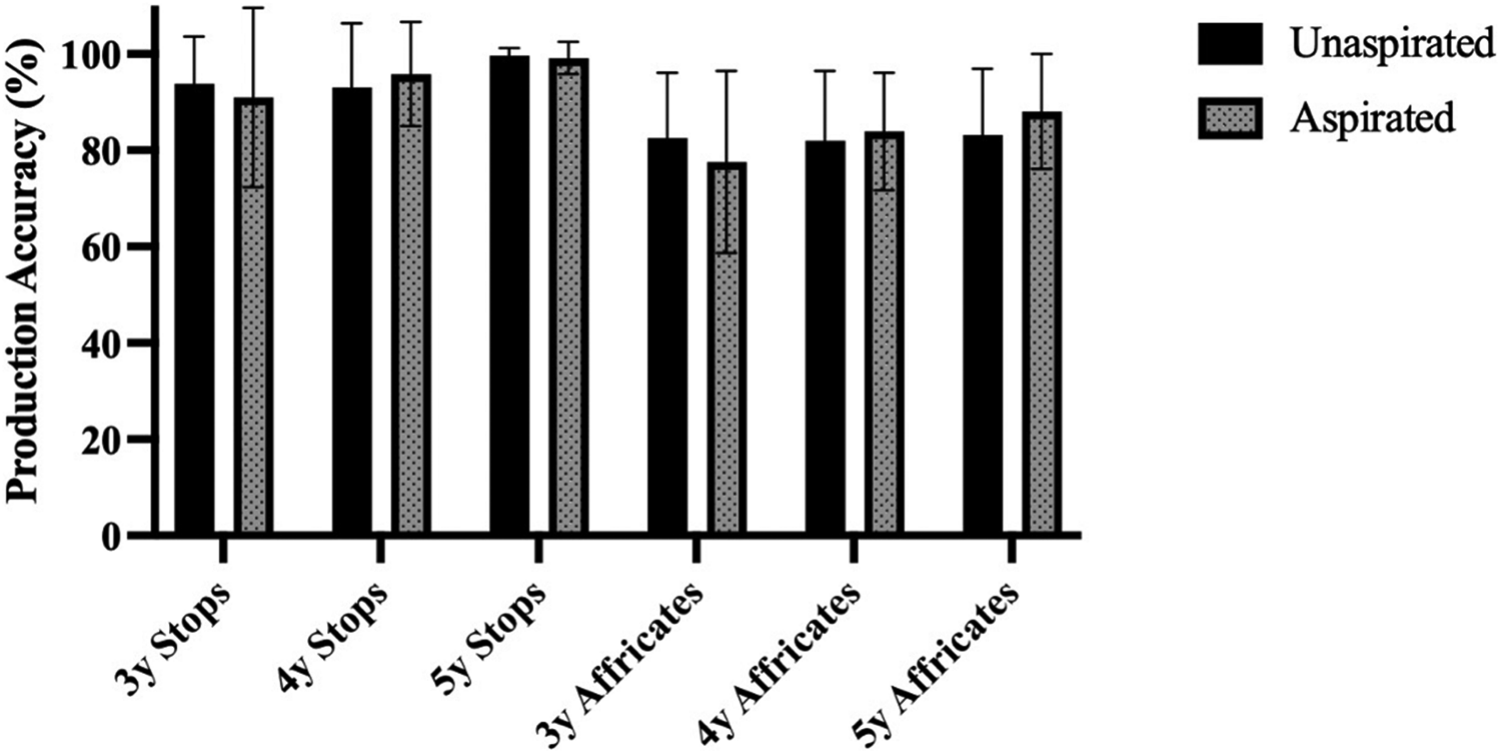
Accuracy of producing aspirated/unaspirated consonants (stops and affricates) in children. Error bars represent ± 1 standard deviation. 3y = 3-year-old group, 4y = 4-year-old group, 5y = 5-year-old group.

To explore factors that may affect children's production performance on consonant aspiration, this study analyzed their ability to produce aspirated and unaspirated stops and affricates across different age groups. Figure 6 presents the production accuracy of children for these consonant types. The accuracy of unaspirated stops for 3-, 4-, and 5-year-olds was 93.8% (SD = 9.9%), 93.1% (SD = 13.3%), 99.7% (SD = 1.5%), respectively, while the accuracy for aspirated stops was 91.0% (SD =18.6%), 95.8% (SD = 10.8%), 99.2% (SD = 3.4%), respectively. The difference between aspirated and unaspirated stops was minimal. Notably, 3-year-olds already demonstrated high accuracy for aspirated/unaspirated stops, and by age 5, their performance approached ceiling levels (over 99%). For affricates, production accuracy was 82.5% (SD = 13.7%), 82.0% (SD = 14.5%), 83.2% (SD = 13.8%) for unaspirated ones, and 77.6% (SD = 18.9%), 83.9% (SD = 12.2%), 88.1% (SD = 12.0%) for aspirated ones, respectively. No great difference between aspirated and unaspirated affricates was observed. The improvement in the production of affricates was slow and gradual, with unaspirated affricates showing a more noticeable increase. Overall, children's accuracy in producing affricates consistently lagged behind that of stops. To statistically validate the influence of *Child Age*, *MOA* and *Aspiration State* on production accuracy, a GLMM was fitted with these factors and their interaction as fixed effects, along with random intercepts by *participant*. The model revealed significant main effects for *Child Age* and *MOA*, and a significant interaction effect of *Child Age* × *Aspiration State* (shown in Table 4). This confirms that pre-school children exhibit a pronounced advantage in producing aspirated/unaspirated stops compared to affricates, and indicates that production performance improves with age. Notably, the age effect appears to differ between aspirated and unaspirated consonants. Post-hoc analyses of the interaction effects revealed that the production accuracy of aspirated consonants improved with age, with 5-year-old children performing significantly better than 3-year-old children (*p* < .05). In contrast, no clear increase in production accuracy was observed across ages for unaspirated consonants. Additionally, no significant differences were found in the production of aspirated and unaspirated consonants within each age group (*p* > .05).

**Table 4:** The results of generalized linear mixed-effects analysis for children's production accuracy (*p < .05, **p < .01, ***p < .001).

Fixed Effects	*χ* ^2^	Df	*p*	
(Intercept)	56.180	1	<.001	***
Child age	6.379	2	.041	*
Aspiration state	0.043	1	.836	
MOA	67.396	1	<.001	***
Aspiration state: MOA	0.186	1	.666	
Aspiration state: Child age	7.823	2	.020	*
MOA: Child age	0.867	2	.648	
Aspiration state: MOA: Child age	2.520	2	.284	

To gain a deeper understanding of the common patterns in children's misarticulations, an error analysis was conducted to identify the prevalence of different pronunciation error types (shown in Table 5). Overall, the most common errors involved substitutions of POA and confusion of aspiration, particularly fronting (e.g., /t/ for /k/) and deaspiration (e.g., /k^h^/ for /k/), accounting for over 20% of errors. Other frequent errors included affrication (e.g., /tɕ/ for /t/), aspiration (e.g., /t^h^/ for /t/), and palatalization (e.g., /ʦ^h^/ for /tɕ^h^/), each comprising more than 10% of errors. Backing errors were the least frequent.

**Table 5:** The percentage of various error types in all incorrect productions. 3y = 3-year-old group, 4y = 4-year-old group, 5y = 5-year-old group.

	Deaspiration	Aspiration	Affrication	Stopping	Frication	Fronting	Palatalization	Backing	Consonant deletion
3 years	16.3%	2.7%	10.3%	5.4%	3.1%	11.3%	10.0%	0.5%	3.2%
4 years	2.7%	5.0%	4.1%	0.0%	0.0%	10.4%	0.9%	0.9%	0.9%
5 years	1.4%	5.9%	0.0%	1.4%	0.5%	1.4%	1.7%	0.0%	0.0%
All	20.6%	13.6%	14.4%	6.8%	3.6%	23.1%	12.6%	1.4%	4.1%

Across age groups, the most common error types varied, with some showing a clear age-related reduction while others persisted. At age 3, children exhibited the most diverse and the highest proportion of errors, with common issues in deaspiration, fronting, palatalization, and affrication (over 10%), followed by stopping, frication (e.g., /h/ for /k^h^/), consonant deletion, and aspiration (e.g., /p^h^/ for /p/) errors. 4-year-olds still exhibited fronting errors, followed by aspiration and affrication errors, but the variety and proportion of mistakes decreased compared to younger children. Notably, aspirated sound errors increased in this group. By age 5, errors in frication, backing, consonant deletion, and frication were nearly eliminated. However, aspiration errors persisted at levels similar to those of 4-year-olds and remained higher than at age 3.

## 4 Study 3: association between perception and production of consonant aspiration

Study 3 combined the results of consonant aspiration perception and production of the 93 children, to explore the potential relationship between aspiration perception and production.

### 4.1 Results

**Figure 7:**
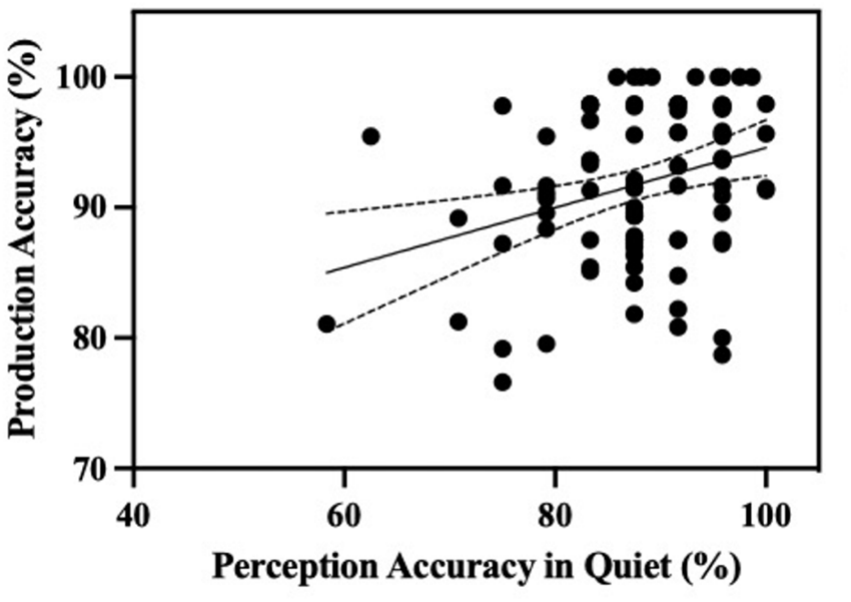
Scatter plots of children's perception in the quiet condition and production.

To explore the relationship between children's perception accuracy in the quiet condition and their production accuracy of consonant aspiration, this study conducted simple linear regression analyses with perception accuracy in the quiet condition serving as the predictor variable. The regression model revealed that perception accuracy significantly predicted children's production accuracy (*β* = 0.251, *SE* = 0.083, *t* = 3.016, *p* = .003), accounting for 19.1% of the variance (*R*^2^ = 0.191). These findings suggest that higher perception accuracy in the quiet condition is associated with better production accuracy for consonant aspiration, as can be seen in Figure 7.

## 5 Discussion

### 5.1 Children's acquisition of consonant aspiration

This study investigates Mandarin-speaking children's acquisition of aspirated/unaspirated consonants from the perspective of perception and production and its association. It is also conducted to document the developmental changes in the perception of aspirated/unaspirated consonants in noise. Overall, the results revealed that children aged 5 have not reached adult levels yet in either perceiving (in quiet/noisy conditions) or producing these consonants.

Under the quiet condition, 5-year-olds demonstrated a robust ability to perceive aspirated/unaspirated consonants, in other words, they were able to distinguish the phonemic features associated with aspiration, although their performance was not entirely on par with adults. This finding is consistent with previous findings by Wong et al. (10), who observed a continuous improvement from the preschool years, with average accuracies of 85%–90% by age 4 and over 90% by age 5. In this study, we employed a 2-AFC task augmented with picture cues. The hypothesis that top-down lexical processing facilitates perceptual judgment (64, 65), suggests that the inclusion of pictures corresponding to word stimuli might enhance perceptual performance. However, our results showed comparable accuracy levels to those observed in Wong et al. (10)'s study, which used a simple discrimination task without such lexical cues. This suggests that at the preschool stage, children's phoneme perception abilities may not be significantly influenced by lexical knowledge or task complexity. Additionally, the slower development of consonant aspiration perception under the quiet condition in our study compared to Liu et al. (9) was expected, given differences in stimulus presentation. In contrast, under the noisy condition, children across all age groups performed significantly worse than in the quiet condition, failing to achieve adult-like proficiency. This highlights the disruptive impact of noise on children's speech perception, an issue that will be discussed later.

Regarding production, children displayed a developmental pattern similar to their perceptual abilities under quiet conditions. Although 5-year-olds performed well, their accuracy remained notably lower than adults. Notably, production accuracy slightly increased with age and was relatively stable across the child age groups. 3-year-olds already demonstrated strong production skills, with accuracy rates exceeding 85%, while 5-year-olds reached over 90%, reflecting a high level of proficiency. These findings suggest that, for Mandarin-speaking children, production accuracy for aspirated and unaspirated consonants develops steadily from an early age. The high production accuracy observed, particularly in younger children, might partially be attributed to the evaluation method used in this study. Native speakers’ perceptual evaluation, while thorough, may involve some subjective judgment. It is noteworthy that formant transitions, which reflect the rapid vocal tract changes following consonant release, play a significant role in consonant perception, alongside VOT cues (66). Although raters were instructed to assess consonants independently of the subsequent vowel, the formant transition could have still influenced auditory judgments. Thus, it is reasonable that the current study found children's production abilities emerge earlier than previously observed based on objective VOT measures (30, 31, 33). These results also underscore the importance of considering evaluation methods when interpreting early production abilities in children and suggest that future studies should incorporate both subjective judgments and objective measurements for a more comprehensive assessment.

Error analysis in this study revealed a decrease in both the percentage of errors and types of errors with increasing age, indicating that children refine their motor speech abilities as they anatomically and physiologically mature (33, 34). As shown in the results, 3-year-olds often substituted unaspirated sounds for aspirated sounds, suggesting they have not yet mastered the fine temporal coordination required to delay laryngeal vibration relative to oral closure release. By age 4, deaspiration errors significantly decreased, but children still occasionally confused unaspirated sounds with aspirated sounds until age 5, indicating an ongoing development of aspiration contrast. In terms of MOA, affrication errors were observed in 3- to 4-year-olds, consistent with previous research (63). Regarding POA, fronting errors were also common in 3- to 4-year-olds, likely due to the later maturation of motor control for posterior articulators (e.g., velar) (39). In addition, 3-year-olds also exhibit some palatalization errors, particularly when followed by front vowels. For example, /ʦ/ followed by /ɨ/ is often replaced by /tɕ/, resulting in mispronunciations such as *zi4 mu3*/tsɨ mu/ (‘字母letter’) be pronounced as /tɕɨ mu/. This issue arises from inaccurate tongue positioning during articulation, like retraction or arching against the hard palate, leading to palatalization errors (67). The prevalence of these production errors during the early stages of aspiration acquisition highlights the importance of timely intervention, particularly for children with speech development challenges, such as those using hearing aids or cochlear implants.

The present findings reveal a correlation between children's perception in the quiet condition and production of consonant aspiration. This differs from previous findings that showed little or no correlation between perception and production abilities (68, 69). For instance, Newman (68), in a study with a small sample size of 25 participants, observed no correlation between perceptual prototypes and the mean VOT values produced for plosives, except for /p^h^a/. This limited finding could be attributed to the small sample size and the narrow focus on specific consonants. In contrast, our study, which involves a larger sample and a more comprehensive investigation of six pairs of aspirated and unaspirated consonants in Mandarin, provides more robust evidence that consonant perception and production are interconnected. Specifically, our results show that children's ability to perceive aspirated and unaspirated consonants predicts their accuracy in producing these sounds.

This supports the view that perception and production are interdependent in speech acquisition, as also observed in studies by Edwards (70) on children's specific phonemic contrasts (e.g., fricatives) in English and by Levy et al. (71) on adults’ vowel perception and production in second language acquisition. Furthermore, research on language acquisition suggests that early perceptual experiences map onto speech production, helping shape articulatory patterns (72–77). Studies on segmental acquisition suggest that phonological knowledge may develop from lexical contexts rather than direct exposure to abstract phonemic features, with phoneme categories developing alongside their use in production and production playing a role in shaping perception (55, 78–80). These findings suggest a synergistic development of phoneme perception and production skills, where each domain influences the other, a pattern that is further corroborated by our study. By concentrating on Mandarin aspirated and unaspirated consonants, which play a pivotal role in phonemic differentiation, our research underscores the interactive and interdependent relationship between perception and production in acquiring these contrasts. This underscores the significance of considering both perception and production factors when examining early phonemic feature development.

While our analysis of substantial data confirms the hypothesis of an association between children's speech perception and production, the variance explained by the perception accuracy is small (*R*^2^ = 0.191), indicating this link may be rather loose. This finding aligns with previous research suggesting that the developmental trajectories for the perception and production of phonemic contrasts may exhibit a dynamic and interactive path rather than a strictly linear progression (54, 55, 78). Although our analysis does not directly assess non-linear developmental patterns, these previous findings provide a valuable perspective on the complexity of speech acquisition. Moreover, this suggests that additional factors, beyond perception ability, contribute to children's development of accurate consonant production. Potential contributors could include individual differences in motor development, individual variability in cognitive skill development, differences in language exposure, and the quality of education in school and family settings (55, 76). Future studies should explore these factors in greater detail to better understand the influence on children's acquisition of consonant aspiration.

### 5.2 Factors affecting children's perception and production of consonant aspiration

In this study, noise is an important factor that significantly affects children's consonant aspiration perception. The SSN used served as an effective energetic masker, spectrally overlapping with the speech signal and causing a masking effect on the perceptual distinctions between aspirated and unaspirated consonants. However, adults’ perception accuracy of aspirated/unaspirated consonants in the noisy condition was similar to that in the quiet condition, with nearly 100% accuracy. This differs from the findings of Winn et al. (17), which showed that adults’ categorical perception of the VOT continuum was affected under SSN at 0 dB SNR and other challenging auditory conditions. The discrepancy may stem from our focus on children's recognition of the aspirated/unaspirated contrast in minimal pairs, rather than on categorical perception of continuous VOT changes. Additionally, the simplicity of the picture selection task used in this study may have contributed to the ceiling effect observed in adults. In contrast, children nearly 6 years (with a maximum age of 5;11 in this study) old had not yet fully developed their perception ability in the noisy environment. 3- to 5-year-old children's auditory systems, which are still developing, are more susceptible to the adverse effects of noise, as their ability to segregate and process speech signals in complex auditory environments matures over a longer developmental trajectory, spanning over 10 years (10, 13).

Furthermore, a comprehensive analysis of children's perceptual performance indicates that, during the preschool years, the perception of unaspirated consonants develops more rapidly than that of aspirated consonants. When examining perception results under different listening conditions, it becomes evident that children face particular challenges in perceiving aspirated consonants in noisy environments compared to unaspirated ones. While no obvious difference in children's perceived accuracy of aspirated vs. unaspirated consonants was found in a quiet environment, there was a significant difference in noise. The findings suggest that noise can obscure the critical acoustic cues needed to distinguish between aspirated and unaspirated consonants. This reduction in perceptual salience of these cues results in masking effects, where the critical temporal (VOT) and spectral (burst) cues become less accessible to listeners (17, 81). Children's auditory processing abilities are not fully developed until adolescence, typically around ages 10–14, which limits their capacity to filter out noise and focus on speech cues (82). Consequently, younger children find it more challenging to detect and process the subtle cues necessary for distinguishing aspirated from unaspirated consonants in noisy environments. This increased susceptibility to noise suggests that children, especially those with auditory processing disorders or those who use cochlear implants, may benefit from specialized training that improves their auditory sensitivity and ability to detect and utilize these acoustic cues under adverse listening conditions.

MOA is another important factor that affects children's consonant aspiration perception in this study. Children demonstrated generally lower perception accuracy for stops (below 80%), while their performance for affricates was superior (above 80%), with a particular advantage for unaspirated affricates. Our findings indicate that Mandarin-speaking pre-school children have a perceptual advantage for affricates over stops, especially in the perception of unaspirated consonants. Previous research has shown that sibilant fricatives are more resistant to masking due to their higher intensity and longer release compared to stops (27, 83). In noise, the frication cue of affricates is more robust than the VOT cue, which may account for the enhanced perception of affricates (10, 27). However, our results did not fully support earlier observations that MOA advantages are primarily driven by noise resistance, as we did not find a significant *Condition* × *MOA* interaction effect. Notably, our data revealed a specific advantage for unaspirated affricates in resisting noise masking, and showed that the accuracy difference between stops (88.68%) and affricates (84.12%) in the quiet condition was smaller than that observed under the noisy condition (stops 71.84%, affricates 78.86%). Although *Condition* × *MOA* interaction was not observed, there was a trend indicating that this advantage may be modulated by noise in this study. These findings suggest that future studies with larger sample sizes may be needed to further validate and expand upon these results.

Regarding children's production of aspirated and unaspirated consonants, the findings indicate that MOA significantly influenced children's production development, with affricates being more challenging than stops, aligning with previous research (38, 39, 41). This is expected given the greater complexity of affricates articulation patterns. However, no significant differences were observed in children's production abilities between aspirated and unaspirated consonants across all age groups, suggesting that the development of aspirated and unaspirated consonant production in children aged 3–5 progresses nearly concurrently, without a clear developmental sequence. The error analysis further revealed a prevalence of deaspiration errors among 3-year-olds and aspiration errors among 4- and 5-year-olds. These findings indicate that as children grow older, their fine motor coordination for controlling the airflow develops, yet their mastery over aspirated and unaspirated consonants remains a work in progress and is susceptible to confusion (84). Therefore, in this study, children's production accuracy was not significantly influenced by the presence or absence of consonant aspiration.

To conclude, the current study examined the acquisition of consonant aspiration in Mandarin-speaking children aged 3–5, covering the aspiration perception in the quiet and noise conditions, and the aspiration production. Firstly, the findings indicated a correlation between perception and production of consonant aspiration in children, although it is weak and observed only in the quiet condition, where perception accuracy is positively correlated with production accuracy. Secondly, children's perception and production abilities of aspirated and unaspirated consonants improved with age. By age 5, children had largely developed production skills and perception skills in the quiet condition (over 90% accuracy), although they still lag behind adults. Conversely, even by the age of 5;11, perceiving aspirated/unaspirated consonants in noise remains challenging. Last but not least, besides age, factors like aspiration state and MOA significantly influenced children's acquisition of aspirated/unaspirated consonants in Mandarin were observed. Noise weakened the children's ability to perceive aspirated/unaspirated consonants as well, especially the aspirated stops.

### 5.3 Clinical implications

This study to some extent provides the clinical guidelines for evaluating and training children with difficulties in perceiving and producing consonant aspiration. The current findings could serve as a benchmark for typically developed Mandarin-speaking children. By understanding the acquisition of aspirated/unaspirated consonants in different age groups, clinicians can tailor individualized approaches to address specific challenges. Last but not least, the study suggests that the development of children's ability to perceive and produce aspirated/unaspirated consonants is interdependent and progresses through continuous interaction, which highlights the importance of integrating both perception and production during speech interventions to ensure the comprehensive support for children's speech development.

### 5.4 Limitations and future direction

The findings of this study are subject to certain limitations. The production task results are based on researchers’ judgment, which introduces a degree of subjectivity. Future research should incorporate acoustic analysis of children's speech to provide a more objective assessment of their acquisition of aspirated and unaspirated consonants, such as VOT values. Additionally, expanding child participants to a younger age range in future studies would facilitate a more comprehensive tracking of developmental milestones, as the current study did not observe a clear watershed in perception and production abilities among 3- to 5-year-olds under the noisy condition. Finally, potential factors influencing the development of children's speech perception and production skills should be explored in more depth in future research.

## Data Availability

The raw data supporting the conclusions of this article will be made available by the authors, without undue reservation.
