# Supplementary material for: Consonant aspiration in Mandarin-speaking children: a developmental perspective from perception and production
Source: Front Pediatr. 2025 Jan 7;12:1465454. doi: 10.3389/fped.2024.1465454 (PMC11776868; doi:10.3389/fped.2024.1465454)
Supplement: Supplementary file 3 [file Table3.docx]

One-sample t-tests for Figure 2 (perception)

|  | Perception Condition | Age Group | Mean | SD | *p* |
| --- | --- | --- | --- | --- | --- |
| 1 | Noise | 3y | 0.721 | 0.106 | < .0001 |
| 2 | Noise | 4y | 0.775 | 0.120 | < .0001 |
| 3 | Noise | 5y | 0.764 | 0.074 | < .0001 |
| 4 | Noise | Adults | 0.996 | 0.013 | < .0001 |
| 5 | Quiet | 3y | 0.832 | 0.085 | < .0001 |
| 6 | Quiet | 4y | 0.86 | 0.108 | < .0001 |
| 7 | Quiet | 5y | 0.903 | 0.063 | < .0001 |
| 8 | Quiet | Adults | 1 | 0 | NA |

Summary Table**:** Multiple comparisons with “Tukey” adjustments on perception accuracy of groups in Figure 2

| Contrast | *β* | SE | df | *t* | *p* |
| --- | --- | --- | --- | --- | --- |
| Noise Adults – Quiet Adults | -0.004 | 0.021 | 111 | 11.076 | 1.000 |
| Noise Adults – Noise 3y | 0.274 | 0.025 | 189 | 6.610 | <.0001 |
| Noise Adults – Quiet 3y | 0.164 | 0.025 | 189 | 8.953 | <.0001 |
| Noise Adults – Noise 4y | 0.221 | 0.025 | 189 | 5.520 | <.0001 |
| Noise Adults – Quiet 4y | 0.136 | 0.025 | 189 | 9.242 | <.0001 |
| Noise Adults – Noise 5y | 0.232 | 0.025 | 189 | 3.708 | <.0001 |
| Noise Adults – Quiet 5y | 0.093 | 0.025 | 189 | 11.244 | 0.0066 |
| Quiet Adults – Noise 3y | 0.279 | 0.025 | 189 | 6.778 | <.0001 |
| Quiet Adults – Quiet 3y | 0.168 | 0.025 | 189 | 9.123 | <.0001 |
| Quiet Adults – Noise 4y | 0.225 | 0.025 | 189 | 5.689 | <.0001 |
| Quiet Adults – Quiet 4y | 0.140 | 0.025 | 189 | 9.408 | <.0001 |
| Quiet Adults – Noise 5y | 0.236 | 0.025 | 189 | 3.874 | <.0001 |
| Quiet Adults – Quiet 5y | 0.097 | 0.025 | 189 | -6.674 | 0.0036 |
| Noise 3y – Quiet 3y | -0.111 | 0.017 | 111 | -2.499 | <.0001 |
| Noise 3y – Noise 4y | -0.054 | 0.022 | 189 | -6.421 | 0.2023 |
| Noise 3y – Quiet 4y | -0.138 | 0.022 | 189 | -1.925 | <.0001 |
| Noise 3y – Noise 5y | -0.043 | 0.022 | 189 | -8.211 | 0.5354 |
| Noise 3y – Quiet 5y | -0.181 | 0.022 | 189 | 2.632 | <.0001 |
| Quiet 3y – Noise 4y | 0.057 | 0.022 | 189 | -1.290 | 0.151 |
| Quiet 3y – Quiet 4y | -0.028 | 0.022 | 189 | 3.084 | 0.9018 |
| Quiet 3y – Noise 5y | 0.068 | 0.022 | 189 | -3.202 | 0.0474 |
| Quiet 3y – Quiet 5y | -0.071 | 0.022 | 189 | -5.180 | 0.0337 |
| Noise 4y – Quiet 4y | -0.085 | 0.016 | 111 | 0.518 | <.0001 |
| Noise 4y – Noise 5y | 0.011 | 0.022 | 189 | -5.815 | 0.9996 |
| Noise 4y – Quiet 5y | -0.128 | 0.022 | 189 | 4.375 | <.0001 |
| Quiet 4y – Noise 5y | 0.096 | 0.022 | 189 | -1.957 | 0.0005 |
| Quiet 4y – Quiet 5y | -0.043 | 0.022 | 189 | -8.109 | 0.5134 |
| Noise 5y – Quiet 5y | -0.139 | 0.017 | 111 | 11.076 | <.0001 |

Pairwise comparisons on perception accuracy of Condition × Aspiration State interaction in Table 3.

| Contrast | *β* | SE | *z* | *p* |
| --- | --- | --- | --- | --- |
| Nois Unaspirated – Quiet Aspirated | -0.071 | 0.114 | -4.313 | 0.0001 |
| Noise Unaspirated – Noise Aspirated | 0.068 | 0.116 | 4.153 | 0.0002 |
| Noise Unaspirated – Quiet Aspirated | -0.082 | 0.114 | -4.952 | <.0001 |
| Quiet Unaspirated – Noise Aspirated | 0.140 | 0.117 | 8.466 | <.0001 |
| Quiet Unaspirated – Quiet Aspirated | -0.011 | 0.113 | -0.639 | 0.9194 |
| Noise Aspirated – Quiet Aspirated | -0.150 | 0.115 | -9.105 | <.0001 |

Pairwise comparisons on perception accuracy of MOA × Aspiration State interaction in Table 3.

| Contrast | *β* | SE | *z* | *p* |
| --- | --- | --- | --- | --- |
| Unaspirated Affricate – Aspirated Affricate | 0.053 | 0.114 | 3.195 | 0.0080 |
| Unaspirated Affricate – Unaspirated Stop | 0.082 | 0.115 | 4.952 | <.0001 |
| Unaspirated Affricate – Aspirated Stop | 0.087 | 0.115 | 5.271 | <.0001 |
| Aspirated Affricate – Unaspirated Stop | 0.029 | 0.115 | 1.757 | 0.2952 |
| Aspirated Affricate – Aspirated Stop | 0.034 | 0.114 | 2.076 | 0.1619 |
| Unaspirated Stop – Aspirated Stop | 0.005 | 0.116 | 0.319 | 0.9887 |

Pairwise comparisons on perception accuracy of Condition × Aspiration State × MOA interaction in Table 3.

| Contrast | *β* | SE | *z* | *p* |
| --- | --- | --- | --- | --- |
| Noise Unaspirated Affricate – Quiet Unaspirated Affricate | -0.033 | 0.157 | -1.431 | 0.8429 |
| Noise Unaspirated Affricate – Noise Aspirated Affricate | 0.118 | 0.163 | 5.045 | <.0001 |
| Noise Unaspirated Affricate – Quiet Aspirated Affricate | -0.046 | 0.158 | -1.958 | 0.5115 |
| Noise Unaspirated Affricate – Noise Unaspirated Stop | 0.119 | 0.163 | 5.12 | <.0001 |
| Noise Unaspirated Affricate – Quiet Unaspirated Stop | 0.011 | 0.160 | 0.452 | 0.9998 |
| Noise Unaspirated Affricate – Noise Aspirated Stop | 0.139 | 0.162 | 5.948 | <.0001 |
| Noise Unaspirated Affricate – Quiet Aspirated Stop | 0.002 | 0.160 | 0.075 | 1.0000 |
| Quiet Unaspirated Affricate – Noise Aspirated Affricate | 0.151 | 0.163 | 6.476 | <.0001 |
| Quiet Unaspirated Affricate – Quiet Aspirated Affricate | -0.012 | 0.158 | -0.527 | 0.9995 |
| Quiet Unaspirated Affricate – Noise Unaspirated Stop | 0.153 | 0.163 | 6.551 | <.0001 |
| Quiet Unaspirated Affricate – Quiet Unaspirated Stop | 0.044 | 0.160 | 1.882 | 0.5635 |
| Quiet Unaspirated Affricate – Noise Aspirated Stop | 0.172 | 0.162 | 7.379 | <.0001 |
| Quiet Unaspirated Affricate – Quiet Aspirated Stop | 0.035 | 0.159 | 1.506 | 0.8043 |
| Noise Aspirated Affricate – Quiet Aspirated Affricate | -0.163 | 0.162 | -7.003 | <.0001 |
| Noise Aspirated Affricate – Noise Unaspirated Stop | 0.002 | 0.165 | 0.075 | 1.0000 |
| Noise Aspirated Affricate – Quiet Unaspirated Stop | -0.107 | 0.165 | -4.593 | 0.0001 |
| Noise Aspirated Affricate – Noise Aspirated Stop | 0.021 | 0.164 | 0.904 | 0.9857 |
| Noise Aspirated Affricate – Quiet Aspirated Stop | -0.116 | 0.163 | -4.97 | <.0001 |
| Quiet Aspirated Affricate – Noise Unaspirated Stop | 0.165 | 0.163 | 7.078 | <.0001 |
| Quiet Aspirated Affricate – Quiet Unaspirated Stop | 0.056 | 0.160 | 2.409 | 0.2383 |
| Quiet Aspirated Affricate – Noise Aspirated Stop | 0.184 | 0.162 | 7.906 | <.0001 |
| Quiet Aspirated Affricate – Quiet Aspirated Stop | 0.047 | 0.159 | 2.033 | 0.4603 |
| Noise Unaspirated Stop – Quiet Unaspirated Stop | -0.109 | 0.164 | -4.668 | 0.0001 |
| Noise Unaspirated Stop – Noise Aspirated Stop | 0.019 | 0.164 | 0.828 | 0.9915 |
| Noise Unaspirated Stop – Quiet Aspirated Stop | -0.118 | 0.164 | -5.045 | <.0001 |
| Quiet Unaspirated Stop – Noise Aspirated Stop | 0.128 | 0.165 | 5.497 | <.0001 |
| Quiet Unaspirated Stop – Quiet Aspirated Stop | -0.009 | 0.162 | -0.376 | 0.9999 |
| Noise Aspirated Stop – Quiet Aspirated Stop | -0.137 | 0.162 | -5.873 | <.0001 |

Pairwise comparisons on perception accuracy of Child Age × Aspiration State interaction in Table 4.

| Contrast | *β* | SE | *z* | *p* |
| --- | --- | --- | --- | --- |
| 3y Unaspirated – 4y Unaspirated | -0.6572 | 0.221 | -2.979 | 0.034 |
| 3y Unaspirated – 5y Unaspirated | -0.6413 | 0.225 | -2.846 | 0.050 |
| 3y Unaspirated – 3y Aspirated | 0.1172 | 0.184 | 0.638 | 0.988 |
| 3y Unaspirated – 4y Aspirated | -0.0821 | 0.217 | -0.378 | 0.999 |
| 3y Unaspirated – 5y Aspirated | -0.0716 | 0.222 | -0.322 | 0.100 |
| 4y Unaspirated – 5y Unaspirated | 0.0159 | 0.225 | 0.071 | 1.000 |
| 4y Unaspirated – 3y Aspirated | 0.7744 | 0.220 | 3.518 | 0.006 |
| 4y Unaspirated – 4y Aspirated | 0.5751 | 0.186 | 3.090 | 0.025 |
| 4y Unaspirated – 5y Aspirated | 0.5855 | 0.224 | 2.618 | 0.093 |
| 5y Unaspirated – 3y Aspirated | 0.7585 | 0.224 | 3.379 | 0.010 |
| 5y Unaspirated – 4y Aspirated | 0.5592 | 0.224 | 2.497 | 0.125 |
| 5y Unaspirated – 5y Aspirated | 0.5697 | 0.191 | 2.984 | 0.034 |
| 3y Aspirated – 4y Aspirated | -0.1993 | 0.217 | -0.918 | 0.942 |
| 3y Aspirated – 5y Aspirated | -0.1889 | 0.222 | -0.851 | 0.958 |
| 4y Aspirated – 5y Aspirated | 0.0105 | 0.221 | 0.047 | 1.000 |
