# Supplementary material for: Consonant aspiration in Mandarin-speaking children: a developmental perspective from perception and production
Source: Front Pediatr. 2025 Jan 7;12:1465454. doi: 10.3389/fped.2024.1465454 (PMC11776868; doi:10.3389/fped.2024.1465454)
Supplement: Supplementary file 4 [file Table4.docx]

Summary Table**:** Multiple comparisons with “Tukey” adjustments on production accuracy of groups in Figure 5

| Contrast | *β* | SE | df | *t* | *p* |
| --- | --- | --- | --- | --- | --- |
| Adults – 3y | 0.076 | 0.016 | 109 | 4.891 | <.0001 |
| Adults – 4y | 0.096 | 0.016 | 109 | 6.210 | <.0001 |
| Adults – 5y | 0.073 | 0.016 | 109 | 4.657 | 0.0001 |
| 3y – 4y | 0.020 | 0.014 | 109 | 1.457 | 0.4668 |
| 3y – 5y | -0.003 | 0.014 | 109 | -0.228 | 0.9958 |
| 4y – 5y | -0.023 | 0.014 | 109 | -1.675 | 0.342 |

Pairwise comparisons on production accuracy of Child Age × Aspiration State interaction in Table 5.

| Contrast | *β* | SE | *t* | *p* |
| --- | --- | --- | --- | --- |
| Unaspirated 3y – Aspirated 3y | 0.039 | 0.018 | 2.179 | 0.251 |
| Unaspirated 3y – Unaspirated 4y | 0.006 | 0.027 | 0.220 | 1.000 |
| Unaspirated 3y – Aspirated 4y | -0.017 | 0.027 | -0.629 | 0.989 |
| Unaspirated 3y – Unaspirated 5y | -0.033 | 0.028 | -1.181 | 0.845 |
| Unaspirated 3y – Aspirated 5y | -0.055 | 0.028 | -1.970 | 0.365 |
| Aspirated 3y – Unaspirated 4y | -0.033 | 0.027 | -1.198 | 0.837 |
| Aspirated 3y – Aspirated 4y | -0.056 | 0.027 | -2.048 | 0.321 |
| Aspirated 3y – Unaspirated 5y | -0.072 | 0.028 | -2.576 | 0.110 |
| Aspirated 3y – Aspirated 5y | -0.093 | 0.028 | -3.366 | 0.012 |
| Unaspirated 4y – Aspirated 4y | -0.023 | 0.018 | -1.326 | 0.770 |
| Unaspirated 4y – Unaspirated 5y | -0.039 | 0.028 | -1.408 | 0.722 |
| Unaspirated 4y – Aspirated 5y | -0.061 | 0.026 | -2.204 | 0.243 |
| Aspirated 4y – Unaspirated 5y | -0.016 | 0.025 | -0.566 | 0.993 |
| Aspirated 4y – Aspirated 5y | -0.037 | 0.028 | -1.361 | 0.480 |
| Unaspirated 5y – Aspirated 5y | -0.022 | 0.018 | -1.213 | 0.830 |
